# Insights into the Formation of Ternary Complexes Among Wheat Starch, Lauric Acid and Protein: Effects of Plasma Pretreatment Times and Protein Types

**DOI:** 10.3390/foods14111922

**Published:** 2025-05-28

**Authors:** Bin Niu, Ziyu Wang, Yizhe Yan

**Affiliations:** 1 College of Food Science and Technology, Henan Agricultural University, Zhengzhou 450000, China; niubin@henau.edu.cn; 2 College of Food and Bioengineering, Zhengzhou University of Light Industry, Zhengzhou 450001, China; 18339162669@163.com

**Keywords:** atmospheric cold plasma, wheat starch-lauric acid-protein complexes, formation, pretreatment times, protein types

## Abstract

Starch-lipid-protein ternary complexes have attracted more attention, and physical processing is gradually being applied to their preparation. This study was to understand the effect of atmospheric cold plasma (ACP) pretreatment times (1–4 min) and protein types (whey protein isolate (WPI), soy protein isolate (SPI), and egg white protein isolate (EWP)) on the wheat starch (WS)-lauric acid (LA)-protein ternary complexes. Experimental results indicated that one-minute ACP pretreatment of WS led to the increase in the amylose content to 30.02%, which produced the largest number of WS-LA-protein complexes (CI value of 69.21%, 67.41%, and 62.81% for WS-LA-WPI, WS-LA-SPI, and WS-LA-EWP complexes, respectively), resulting in the most ordered structure and higher enthalpy change. In vitro digestibility results based on starch showed that WS1-LA-protein complexes exhibited the lowest digestibility with the highest resistant starch content of 28.09%, 27.93%, and 27.41% for these three kinds of complexes, respectively. However, when the treatment time for WS was more than 1 min, a downward trend occurred, indicating that ACP pretreatment of WS for 1 min was the most beneficial for forming complexes. PCA results also verified that ACP pretreatment of WS for different times could significantly impact the generation and structure of ternary complexes. Moreover, protein types also affected the formation and physicochemical properties of ternary complexes. Notably, WPI, with the higher emulsifying property, formed a larger number (CI value of 69.21%), more ordered structure (Xv of 10.56%), and higher thermal stability of ternary complexes than SPI and EWP. This study presents a burgeoning technology to regulate the generation, structure, and functional properties of starch-lipid-protein complexes.

## 1. Introduction

In recent years, starch-lipid-protein ternary complexes have been paid more and more attention due to their special structure, significantly affecting the quality of the final food products [1,2]. The starch-lipid-protein complexes were formed by the hydrophobic interactions, van der Waals forces between starch and lipid, and van der Waals forces and hydrogen bonds between starch and protein [3]. They displayed a V-type crystalline structure, while they were more ordered in both short-range and long-range structures and had increased steric hindrance than corresponding starch-lipid binary complexes [4,5]. Moreover, ternary complexes exhibited higher viscosity, better water-soluble and thermostability, and lower digestibility than binary complexes due to the presence of protein [6,7].

As a type of resistant starch (RS5), their formation can lower the digestibility of starch. What’s more, they are expected to be applied to specialty foods due to the water-soluble property. Starch-lipid-protein ternary complexes have been widely studied, and their formation is influenced by many factors, such as starch source, amylose content, degree of polymerization (DP) of amylose, the type of lipid and protein, complexing conditions, and so on. Wheat and maize starches were easier to form a larger number of ternary complexes than potato starch, while waxy maize starch was the least likely to form ternary complexes among these four types of starches [8]. Appropriate debranching treatment could promote the formation of starch-lauric acid-β-lactoglobulin complexes due to the increased content of amylose, while extensive debranching was unfavorable because of the low DP of amylose [9]. Both potato starch and OSA-modified potato starch formed ternary starch-lipid-protein complexes with various monoglycerides and β-lactoglobulin [10], while maize starch did not [11]. Fatty acids with short chains or lower unsaturation were favorable for forming ternary complexes, although their thermostability was relatively poor [6]. Protein (e.g., whey protein isolate) with an isoelectric point lower than 7.0 or with better emulsifying properties was prone to form more starch-lipid-protein ternary complexes [12,13], while endogenous gluten had stronger influences on the ordered degree, pasting, and digestive properties of the wheat starch-oleic acid-protein system compared with whey protein isolate [14]. Moreover, the generation of ternary complexes was also promoted by controlling the processing conditions, such as increasing the complexation temperature and cooling rate [4]. Recently, the formation of ternary complexes regulated by physical field treatment has gradually attracted our attention. Ultrasonic pretreatment of starch with suitable power facilitated the formation of ternary complexes to a certain extent due to the increased amylose content. However, excessive ultrasonic pretreatment showed adverse effects due to the production of more short-chain amylose [15].

As a non-thermal technology, atmospheric cold plasma (ACP) has gained great interest in food processing due to its economical, multi-functional, and environmentally friendly features. It has been proven to be applicable for food sterilization, food modification, nutrient extraction, and so on [16]. The application of ACP in the starch field includes improving starch’s solubility and destroying the chemical bond and crystal structure of native starch [17]. Their wide application in starch modification is due to the generated reactive species, which could cause physical and chemical changes in starch [18]. As we all know, starch consists of amylose and amylopectin. Amylose is a lightly branched polysaccharide mainly consisting of α-1,4 linkage, whereas amylopectin is a highly branched polysaccharide consisting of α-1,4 and α-1,6 linkage [19]. When the high-energy particles (e.g., reactive oxygen species (ROS) and reactive nitrogen species (RNS)) reached the starch, they could decompose the surface of the starch and break α-1,4 or α-1,6 linkages, especially the α-1,6-glycosidic linkages [20]. Therefore, both amylose and amylopectin could depolymerize into short-chain pieces, resulting in an increase in amylose content [17,21]. The depolymerization is affected by the plasma species, formed free radicals, ions concentration, and the properties and morphology of the starch, in which the treatment conditions of plasma are a principal element in modifying starch [22,23]. Therefore, degradative starch with different depolymerization degrees and high amylose content can be obtained by regulating ACP treatment conditions.

On the basis of the previous research, high amylose content is more conducive to forming starch complexes [2]. ACP pretreatment of starch is expected to be applied in improving the construction of ternary complexes. Very recently, we have found that ACP pretreatment of wheat starch for 1 and 2 min could improve the amylose content, thus resulting in the incremental number of ternary complexes among wheat starch, various fatty acids, and β-lactoglobulin and the decrease in their digestibility [20]. Moreover, the property of protein has exhibited significant effects on the formation and property of ternary complexes. Therefore, herein the effect of ACP treatment with different processing times on the formation, long-range or short-range ordered structure, thermal properties, and in vitro digestibility of starch-lipid-protein ternary complexes from wheat starch, lauric acid, and different proteins (whey protein isolate, soy protein isolate, and egg white protein isolate) was investigated. This study extends the application of ACP and provides a new physical treatment method for regulating the formation and property of starch-lipid-protein ternary complexes, which has potential applications in functional foods such as special medical food and low-GI food.

## 2. Materials and Methods

### 2.1. Materials

Wheat starch (WS) was provided by Shandong Qufeng Food Science and Technology Co., Ltd. (Weifang, China), containing 0.4% lipid and 0.3% protein. Lauric acid (LA, C12:0) was purchased from Aladdin Biochemical Technology Co. (Shanghai, China). Whey protein isolate (WPI), soy protein isolate (SPI), and egg white protein (EWP) were purchased from Shanghai Yuanye Bio-Technology Co., Ltd. (Shanghai, China). Porcine pancreatic enzyme (8 × USP, P7545) and amyloglucosidase (260 U/mL, A7095) were provided by Sigma-Aldrich (Shanghai, China). The assay kits used were procured from Megazyme International Ireland Ltd. (Wicklow, Ireland). All chemical reagents employed were of analytical grade.

### 2.2. ACP Treatment of WS

WS slurry (0.2 g/mL) was pretreated by an ACP jet apparatus (Easton Geake Automation Equipment Co., Ltd., Shenzhen, China) at the input power of 750 W, high frequency power of 25 kHz, and working air pressure of 0.18 MPa [20]. The slurry surface was 15 mm away from the plasma probe, and the treatment time was 0, 1, 2, 3, and 4 min, respectively. After centrifuging, freeze-drying, grinding, and sieving, the ACP-modified WS were obtained and named as WS (0–4).

### 2.3. Fabrication of WS-LA-Protein Ternary Complexes

A rapid viscosity analyzer (RVA4500, Perten, Hägersten, Sweden) was used to obtain the WS-LA-protein ternary complexes according to the method of Cai et al. [8]. 2 g of ACP-modified WS, 100 mg of LA, and 200 mg of different proteins (WPI, SPI, and EWP) were weighed in RVA cans, and then 25.7 g of deionized water were added. Standard procedure 1 was performed to produce WS-LA-protein ternary complexes. The resulting paste underwent a series of operations, including freeze-drying, grinding, and sieving, to gain the ternary complexes samples, denoted as WS(0–4)-LA-WPI, WS(0–4)-LA-SPI, and WS(0–4)-LA-EWP.

### 2.4. Amylose Content Analysis

Amylose (AM) content of ACP-modified WS was measured according to our previous method [20].

### 2.5. Complexing Index (CI) Analysis

The CI value of WS-LA-protein ternary complexes was measured according to the method described by Yan et al. [20]. 5.0 g of WS-LA-protein paste obtained by the RVA procedure were mixed with 25 mL of distilled water and vortexed for 1 min. The mixture was centrifuged (1744× *g*, 15 min), and 100 μL of the supernatant was blended with 15 mL of deionized water and 2 mL of an iodine solution (2.0% KI and 1.3% I_2_). The UV absorbance at 620 nm was measured. The corresponding ACP-modified WS subjected to identical RVA procedures was employed as a reference. CI values of different complexes were calculated according to the following Equation (1).

(1) $$\mathrm{CI}\;(\%)=\frac{Abs_{reference}-Abs_{\mathrm{WS}-\mathrm{LA}-\mathrm{protein}}}{Abs_{reference}}\times 100$$

where *Abs_reference_* was the absorbance of the corresponding ACP-modified WS pastes, and *Abs_WS-LA-protein_* was the absorption of the WS-LA-protein ternary complex pastes.

### 2.6. X-Ray Diffraction (XRD)

The XRD patterns of the complexes were determined by an X-ray diffractometer (Empyrean, Malvern Panalytical, Worcestershire, UK) operating at 40 kV and 40 mA with a scanning rate of 2°/min and a step size of 0.02°. The scanning angle ranges from 5° to 35° (2*θ*), and the relative crystallinity of V-type complexes (*Xv*) was obtained according to the following Equation (2).

(2) $$X_{V}\;(\%)=\frac{A_{v}}{A_{c}+A_{a}}\times 100$$

where *Ac* was the crystallization area, *Aa* was the amorphous area, and *Av* was the V-type crystalline area.

### 2.7. Fourier Transform Infrared (FTIR) Spectroscopy

The FTIR patterns of WS-LA-protein complexes were measured by a Fourier transform infrared spectrometer (Vertex 70, Bruker, Karlsruhe, Germany) with the scanning wavelength range of 4000 to 400 cm^−1^, cumulative of 32 scans and resolution of 4 cm^−1^. The FTIR spectra were deconvoluted within the range of 1200–800 cm^−1^ by OMNIC 8.2 software to obtain the absorbance at 1047 cm^−1^ and 1022 cm^−1^ [20].

### 2.8. Differential Scanning Calorimetry (DSC)

A differential scanning calorimeter (Q20, TA, Newcastle, DE, USA) was used to measure the thermodynamic properties of the WS-LA-protein complexes. The obtained WS-LA-protein complexes (3 mg) and distilled water (three times the complexes mass) were equilibrated in an aluminum pan for 24 h at room temperature. Then the aluminum pan was heated from 20 to 120 °C at the rate of 10 °C/min. An empty aluminum pan acted as the reference, and the thermodynamic parameters were analyzed using TA Instruments Universal Analysis 2000 software.

### 2.9. In Vitro Digestibility

The in vitro digestibility of ternary complexes based on starch was measured by the previous method described by Yan et al. [20]. The first was to prepare a mixed enzyme solution: 3 g of porcine pancreatic enzyme was mixed with distilled water (seven times the enzyme mass) and then centrifuged for 20 min at 2725× *g*. The resulting supernatant was taken out and blended with 0.795 mL of amyloglucosidase and 1.755 mL of distilled water, which was the needed solution. The next was the digestibility test, 200 mg of the complexes was weighed and put into 4 mL of NaOAc buffer solution (0.1 mol/L, pH 5.2). The mixture was water-bathed at 37 °C, and 1 mL of mixed enzyme solution was added. After hydrolysis, 0.1 mL of the hydrolyzed solution was inactivated by 4 mL of 70% ethanol and then centrifuged at 2725× *g* for 10 min. The resulting supernatant (0.1 mL) was mixed with GOPOD (3 mL) and colored in the 45 °C water bath for 20 min. The absorbance of samples, standard glucose solution (act as standard), and distilled water (act as blank) at the wavelength of 510 nm was determined. The proportions of rapidly digestible starch (RDS), slowly digestible starch (SDS), and resistant starch (RS) were calculated on the basis of the following Equations (3)–(5).

RDS (%) = 0.9 × (G_20_ − FG) (3)

SDS (%) = 0.9 × (G_120_ − G_20_) (4)

RS(%) = 1 − RDS − SDS(5)

where, G_20_ and G_120_ were representatives of the glucose percentages after enzymatic hydrolysate for 20 min and 120 min, respectively; FG was the percentage of glucose before the enzymatic hydrolysis in the sample.

### 2.10. Statistical Analysis

All of the experiments were repeated three times, and the results were expressed by mean values ± standard deviations. One-way analysis of variance (ANOVA) and post hoc Duncan’s multiple range test (*p* < 0.05) were employed to determine the significant differences between the means by SPSS 26. Principal component analysis and all graphs were produced using Origin 2021.

## 3. Results and Discussion

### 3.1. Amylose Content

The amylose content of WS0 was 28.71% (Table 1), which increased to 30.01% when the WS was treated by atmospheric cold plasma equipment for 1 min. This was because high-energy particles produced by plasma cleaved glycosidic linkages of starch molecules, especially amylopectin (called starch depolymerization), resulting in the generation of more linear starch molecules and increasing amylose content [18]. The depolymerization of amylopectin was confirmed by other studies, which found that tartary buckwheat, banana, pea, taro, and potato starch treated with plasma brought about the decrease in the percentage of long chains (B2 and B3) and the increase in short-medium chains (A and B1) according to branch chain length distribution of amylopectin [24,25,26]. With the extension of plasma processing time, the amylose content decreased to 29.29% for WS2, followed by WS3 (27.71%) and WS4 (27.23%). The reduced amylose content may be due to excessive plasma treatment, which led to the destruction of α-1,4 glucoside bonds in amylose and the formation of soluble sugar (maltose, maltotriose, and maltotetrose), resulting in the decrease in measured amylose content [20].

### 3.2. Complexing Index (CI)

The CI value refers to the amylose content used to form the V-type complexes and is used to evaluate the number of starch-lipid-protein complexes formed [27]. For the WS-LA-WPI complexes (Table 2), the WS0-LA-WPI complex had a CI value of 68.33%, and the highest CI value was given by the WS1-LA-WPI complex (69.21%), indicating the largest number of complexes generated from WS1, LA, and WPI due to the increased amylose content of WS1 (Table 1). With the extension of plasma processing on WS to 4 min, the amount of formed ternary complexes decreased to 67.37%, due to the reduced amylose content. A similar trend was shown in WS-LA-SPI and WS-LA-EWP ternary complexes, while the highest CI value was provided by complexes formed with WS1, and the lowest CI value was provided by WS4 complexes. These results displayed that the number of formed ternary complexes exhibited a positive correlation with amylose content regardless of the type of protein being used, suggesting that amylose content played an important role in complex formation. Moreover, for the same ACP treatment time, the CI value of ternary complexes formed from WPI was higher than that of SPI, followed by EWP; for example, WS1-LA-WPI (69.21%) > WS1-LA-SPI (67.41%) > WS1-LA-EWP (62.81%). The highest CI value meant that WPI facilitated the generation of ternary complexes to a greater extent compared to SPI and EWP, which might be due to the best emulsification ability of WPI [13]. Proteins with better emulsification ability improved the solubility of fatty acids and in turn facilitated the formation of the ternary complexes [5,13].

### 3.3. Crystalline Structure

**Figure 1:**
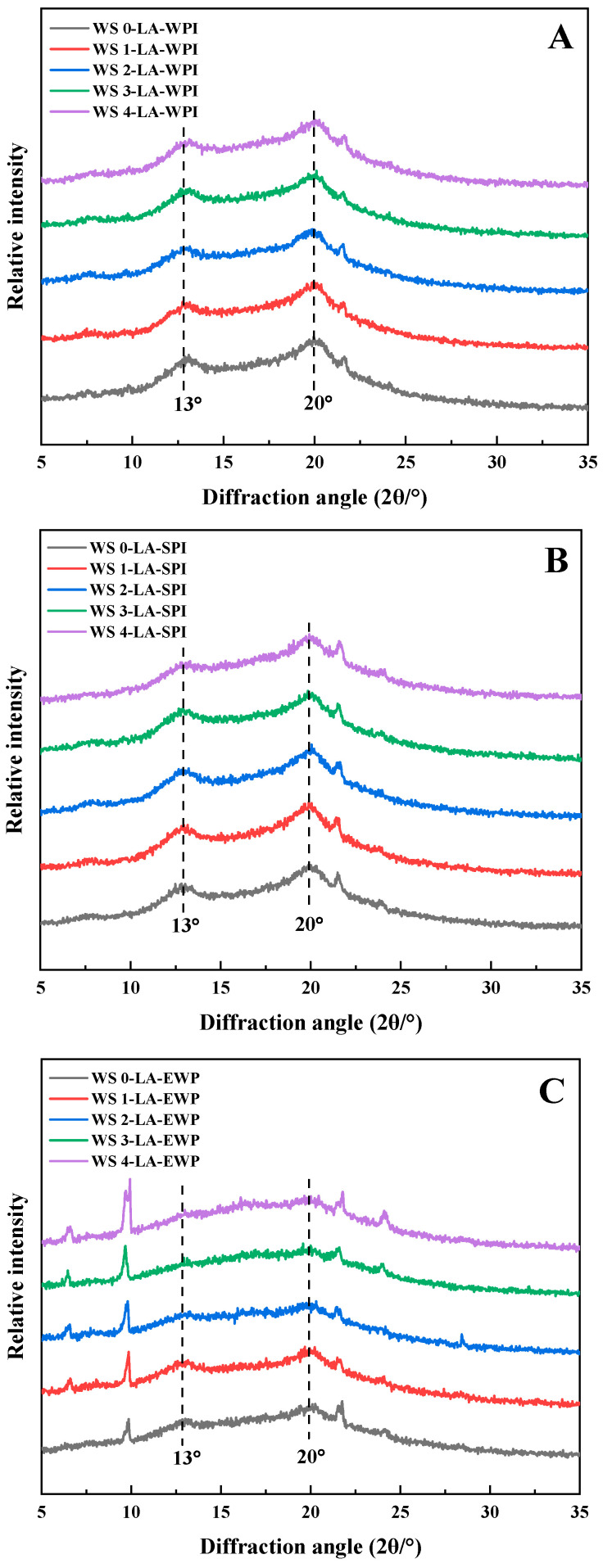
XRD patterns of different WS-LA-protein complexes. (**A**) WS-LA-WPI, (**B**) WS-LA-SPI, (**C**) WS-LA-EWP.

The X-ray diffraction patterns and the relative crystallinity (Xv) of WS-LA-protein ternary complexes are displayed in Figure 1 and Table 1. There were obvious diffraction peaks at 13° and 20° (2*θ*) in diffraction patterns of WS-LA-WPI samples (Figure 1A), representative of V-type crystallites, indicating the formation of WS-LA-WPI ternary complexes [15]. The other small peaks (e.g., the peaks at 6.6°, 9.8°, and 21.3°) came from the uncomplexed LA. The characteristic peaks of V-type crystallites were also presented clearly in the patterns of WS-LA-SPI samples (Figure 1B), meaning the WS-LA-SPI ternary complexes formed when the WS underwent the ACP treatment for 0–4 min. The diffraction peak of uncomplexed LA at around 21.3° seemed more intense than that in WS-LA-WPI ternary complexes, which indicated that WPI can promote more LA participating in the formation of complexes than SPI, and thus more ternary complexes were generated with WPI. For the WS-LA-EWP samples, the characteristic peaks of V-type crystallites at 13° and 20° (2*θ*) could be seen in WS (0–2)-LA-EWP complexes, and they were not evident in the other two samples. What’s more, the other sharp peaks representative of uncomplexed LA were more distinct than WS-LA-WPI and WS-LA-SPI samples, indicating that EWP was not easy to form complexes compared with WPI and SPI, due to their poor emulsifying properties. Similarly, Duan et al. also found that proteins with poorer emulsifying properties are not conducive to the rearrangement of ternary complexes into more ordered structures, which might be manifested in crystalline characteristics [13].

The Xv value of WS-LA-WPI complexes enhanced from 10.18% to 10.59% when the treatment time of ACP rose from 0 min to 1 min (Table 2), signifying that the V-type crystallites were promoted due to the increase in formed complexes. Then the Xv value decreased to 9.72% when the ACP treatment time was 4 min, suggesting that overtreatment of WS led to a reduction in long-range ordered structure because of the lower number of complexes. The same trends are displayed in WS-LA-SPI complexes and WS-LA-EWP complexes (Table 2), showing that WS1 complexes exhibited the highest long-range order because WS1 with the highest AM content was more conducive to forming ternary complexes with LA and protein. Additionally, for the ternary complexes formed by the same WS, the effect of protein on the Xv followed the order of WPI > SPI > EWP. For example, 10.56% of WS1-LA-WPI > 10.13% of WS1-LA-SPI > 9.43% of WS1-LA-EW. The higher Xv value was caused by the larger number of formed ternary complexes due to the better emulsifying property of WPI, followed by SPI and EWP.

### 3.4. Short-Range Ordered Structure

**Figure 2:**
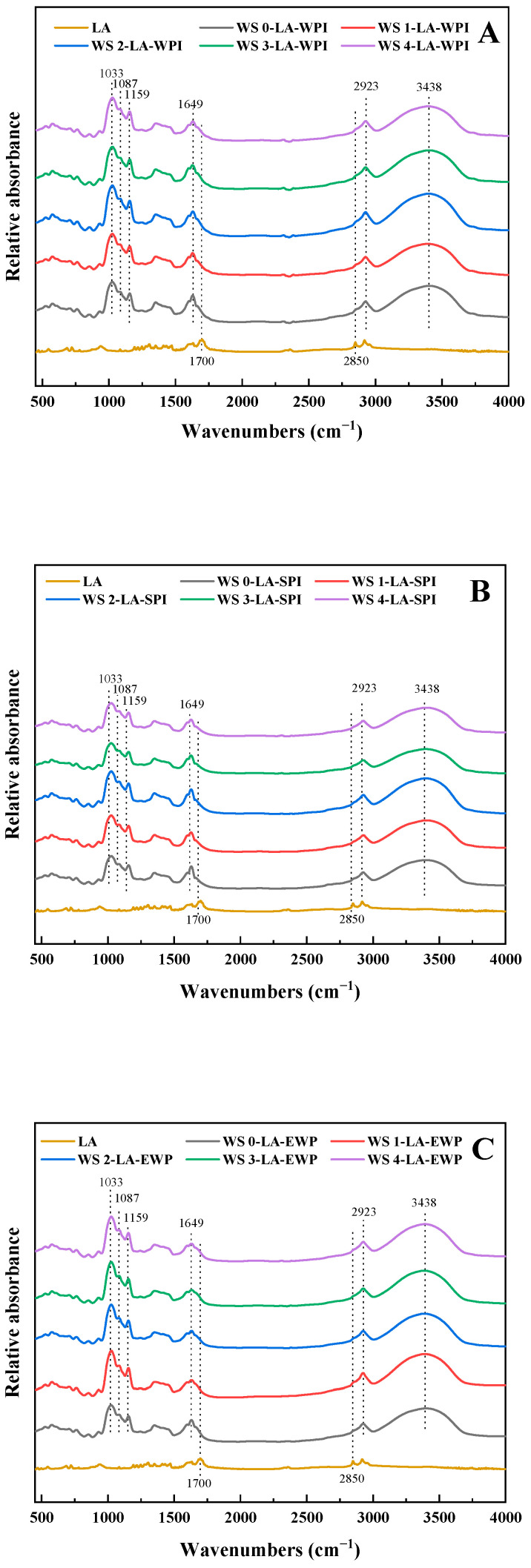
IR spectra of different WS-LA-protein complexes. (**A**) WS-LA-WPI, (**B**) WS-LA-SPI, (**C**) WS-LA-EWP.

The IR spectra can be used to determine the changes in the molecular structure of starch. R_1047/1022_ was the ratio of absorbance at 1047 (representative of the ordered structure of starch) and 1022 cm^−1^ (representative of the amorphous structure of starch), which was always applied to characterize the short-range ordered structure of starch complexes [28]. A higher R_1047/1022_ value represented a more ordered short-range structure of ternary complexes. The IR spectra and corresponding R_1047/1022_ value of WS-LA-protein complexes were shown in Figure 2 and Table 2. As shown in Figure 2, all complexes have characteristic peaks at 3438, 2923, 1649, 1159, 1087, and 1033 cm^−1^. The broad band at 3438 cm^−1^ was attributed to the stretching vibration of O-H in WS [29]. The characteristic peak at 2923 cm^−1^ was attributed to C–H deformation vibration of the glucose element [30]. The peak at 1649 cm^−1^ was related to the bending vibration of O–H in water [31]. The peaks at 1159, 1087, and 1033 cm^−1^ were attributed to the asymmetric C–O–C, C–O, and C–C skeleton stretching vibrations, respectively [32]. There was almost no distinction between WS-LA-protein complexes with diverse WS (treated for 1–4 min), indicating that different ACP treatment times did not bring about the forming of new groups in WS complexes. Free LA displayed two characteristic peaks at 2850 and 1700 cm^−1^, which were attributed to the stretching vibration of the C=O and of the CH_2_ group. However, in IR spectra of the complexes, these two peaks disappeared, which might be because the formation of the complexes causes the protein to wrap around the fatty acid, generating a shielding effect.

As displayed in Table 2, the R_1047/1022_ value for WS-LA-WPI complexes increased from 0.798 to 0.811 when the treatment time of ACP for WS changed to 1 min. The higher R_1047/1022_ value signified a more short-range ordered structure of WS1-LA-WPI because of the formation of more V-type complexes. This improvement indicated that 1 min of ACP treatment on WS could facilitate the generation of ternary complexes because of the increase in amylose content. As the ACP treatment time of WS continued to 2, 3, and 4 min, the R_1047/1022_ value decreased to 0.804, 0.792, and 0.787 for WS2-LA-WPI, WS3-LA-WPI, and WS4-LA-WPI, respectively. The decrease in R_1047/1022_ values manifested the deteriorating short-range order structure of the sample due to the reduction in the formed ternary complexes, in accordance with the CI and XRD results. The reduced number of WS-LA-WPI ternary complexes was caused by the lessened amylose content. WS-LA-SPI and WS-LA-EWP complexes exhibited identical variation tendency compared with WS-LA-WPI complexes, with the highest R_1047/1022_ value provided by WS1-LA-SPI (0.792) and WS1-LA-EWP (0.770), and the lowest R_1047/1022_ value provided by WS4-LA-SPI (0.779) and WS4-LA-EWP (0.748). The above results indicated that WS obtained with 1 min of ACP treatment was more suitable for forming ternary complexes, no matter which protein was used, on account of the highest amylose content (Table 1). Additionally, for the complexes formed from the WS obtained by the same ACP treatment time, the R_1047/1022_ value of WS-LA-WPI complexes was higher than that of WS-LA-SPI complexes, followed by WS-LA-EWP complexes; for example, WS1-LA-WPI (0.811) > WS1-LA-SPI (0.792) > WS1-LA-EWP (0.770), meaning that the WPI was more likely to form ternary complexes than SPI and EWP, due to their better emulsibilities [13].

### 3.5. Thermal Properties

**Figure 3:**
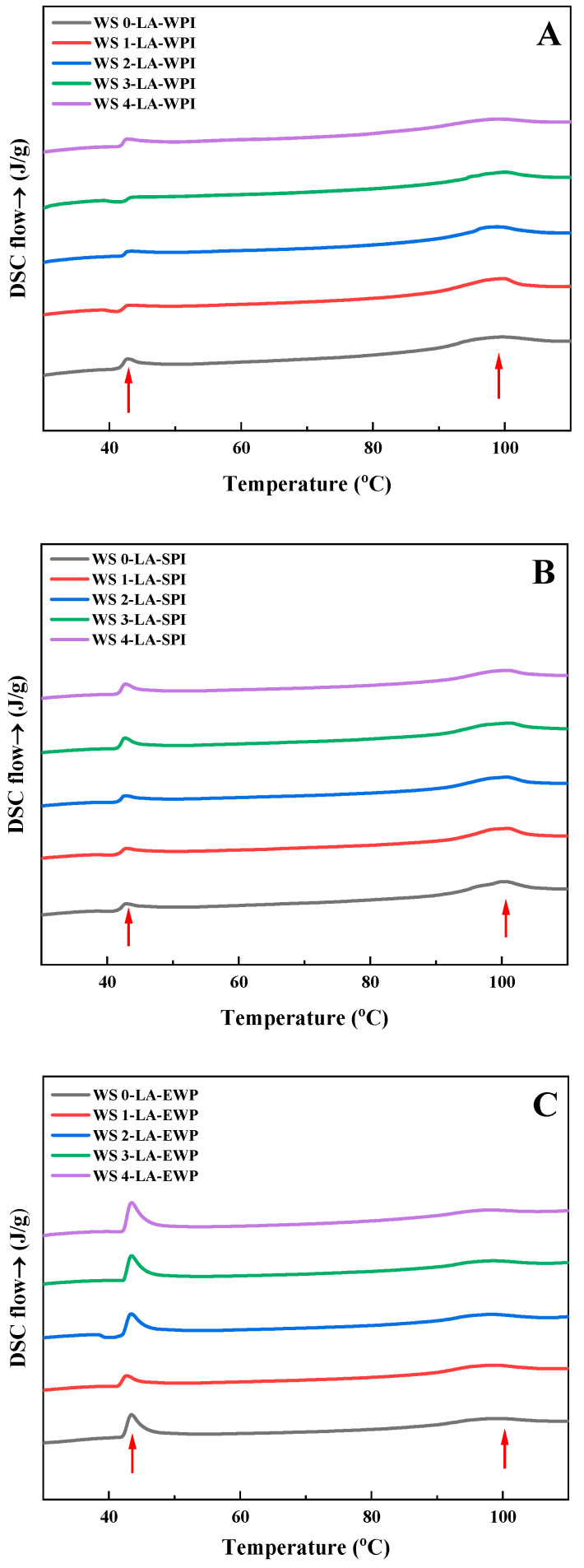
DSC curves of different WS-LA-protein complexes. (**A**) WS-LA-WPI, (**B**) WS-LA-SPI, (**C**) WS-LA-EWP.

The energy variation and the melting of ordered structures of starch complexes were clarified by DSC [20]. DSC curves and thermal transition parameters of WS-LA-protein complexes are displayed in Figure 3 and Table 2. There were two distinct endothermic transitions (indicated by the red arrows) in each DSC curve at around 43 °C and 100 °C, which were ascribed to the melting of uncomplexed LA and WS-LA-protein complexes, respectively [4]. According to Table 2, the ΔH values increased to 4.26 J/g of WS1-LA-WPI complexes compared with WS0-LA-WPI complexes (3.57 J/g), indicating that the formation of WS-LA-WPI complexes was stimulated when the treatment time of ACP for WS augmented from 0 to 1 min, in accordance with the CI, XRD, and FTIR results. As the ACP processing time further increased, the ΔH values declined gradually. For example, the ΔH values for WS2-LA-WPI, WS3-LA-WPI, and WS4-LA-WPI complexes were 3.93, 3.42, and 3.14 J/g, respectively. However, there were no significant distinctions in thermal transition temperature among different WS-LA-WPI complexes, especially for T_o_ and T_p_. Similarly, the ΔH values of WS-LA-SPI and WS-LA-EWP complexes also exhibited the tendency to increase first and then decrease. WS1 is also prone to form WS-LA-SPI and WS-LA-EWP complexes among WS (0–4), with the highest ΔH values of 3.73 J/g for WS1-LA-SPI and 3.13 J/g for WS1-LA-EWP. Moreover, the lowest ΔH values were given by WS4, with WS4-LA-SPI at 3.19 J/g and WS4-LA-EWP at 2.29 J/g. The thermal transition temperatures of WS-LA-SPI and WS-LA-EWP complexes have no evident changes among different WS. However, the T_p_ and ΔH values of WS-LA-WPI complexes using the same kind of WS were higher than WS1-LA-SPI complexes, followed by WS-LA-EWP complexes, explaining that WPI was more likely to make the formed ternary complexes have higher thermal stability compared to SPI and EWP. Moreover, it can be clearly seen that the area and intensity of the melting peak of uncomplexed LA in WS-LA-WPI complexes were lower than that in WS-LA-SPI complexes, followed by WS-LA-EWP complexes (Figure 3). This indicated that free LA present in WS-LA-WPI samples was the least, which meant that a greater amount of LA participated in the generation of the WS-LA-WPI ternary complexes, leading to the larger number of WS-LA-WPI complexes compared with the other two complexes. As we discussed in the XRD section, proteins with better emulsifying properties not only favored the formation of complexes with larger numbers but also made them more ordered, which may lead to higher thermal stability.

### 3.6. In Vitro Digestibility

**Figure 4:**
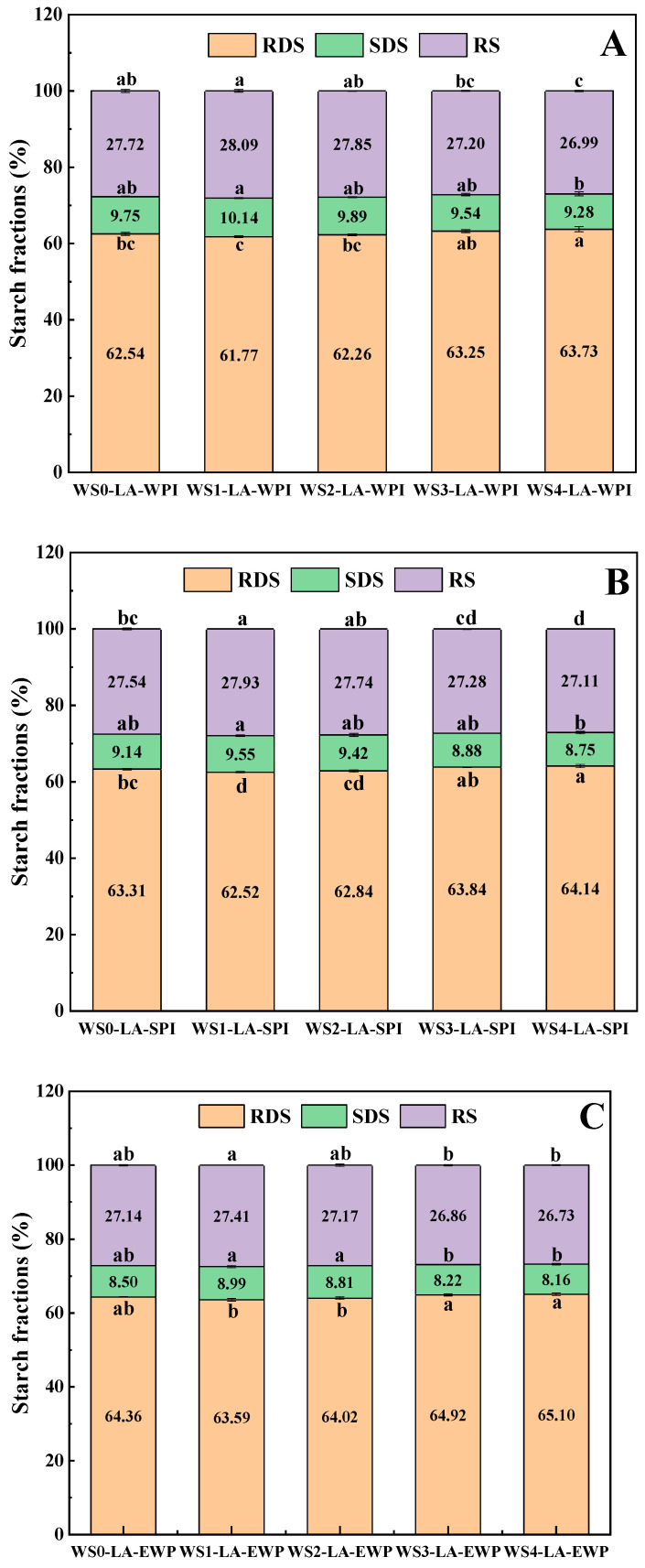
In vitro digestion of different WS-LA-protein complexes. (**A**) WS-LA-WPI, (**B**) WS-LA-SPI, (**C**) WS-LA-EWP. Different letters for RDS, SDS or RS indicate a significant difference (*p* < 0.05).

The in vitro digestibility of starch refers to the evaluation of the rate and degree of starch hydrolysis into glucose by simulating the digestive environment and process of the human small intestine under laboratory conditions, which was important for evaluating the influence of starch on blood glucose levels, the duration of satiety, and glucose metabolism status [33,34]. Rapidly digestible starch (RDS) can be digested rapidly and release glucose immediately, which results in the increase of the postprandial blood glucose concentration and may cause some metabolic chronic diseases that are related to diet, like type 2 diabetes [35]. Slowly digestible starch (SDS) can be digested completely but slowly, which is beneficial to postprandial blood glucose stability [36,37]. Resistant starch (RS) cannot be digested in the healthy human digestive tract, whereas it is degraded by microbial communities through anaerobic fermentation in the colon, which is beneficial for regulating gut microbiota and preventing metabolic diseases [34,38,39]. Thus, the higher the content of SDS and RS, the more conducive to postprandial blood glucose stability. The in vitro digestibility of WS-LA-protein complexes was shown in Figure 4. For WS-LA-WPI complexes, when the WS was treated by ACP for 1 min, the RS and SDS contents increased from 27.72% and 9.75% to 28.09% and 10.14%, respectively, although the significant difference was small. Then the RS and SDS contents decreased to 26.99% and 9.28% with the treatment time of ACP further extending to 4 min. The highest RS and SDS contents of the WS1-LA-WPI sample were due to the largest number of ternary complexes, because they were resistant to enzymic digestion and regarded as one type of RS (RS5) [2]. The structures of V-type complexes were compact and ordered, which resulted in it being difficult for them to act as enzyme substrates [40] and impeded the entrance of hydrolase [41]. In addition, large steric hindrance of ternary complexes may also account for their low digestibility [2]. The subsequent decrease in RS and SDS contents was also caused by the reduction in the number of WS-LA-WPI complexes (Figure 4). The trend of RDS was opposite to that of RS and SDS. The digestibility of WS-LA-SPI and WS-LA-EWP complexes also exhibited the same tendency as WS-LA-WPI complexes. The RS and SDS contents of WS-LA-SPI and WS-LA-EWP complexes increased first and then decreased, with the highest value presented by WS1 complexes, consistent with the changes in the number of ternary complexes. The changes in RDS content showed an opposite pattern to the number of complexes. These results further demonstrated that the forming V-type ternary complexes could reduce starch in vitro digestibility and were beneficial for postprandial blood glucose stability.

### 3.7. Principal Component Analysis (PCA)

**Figure 5:**
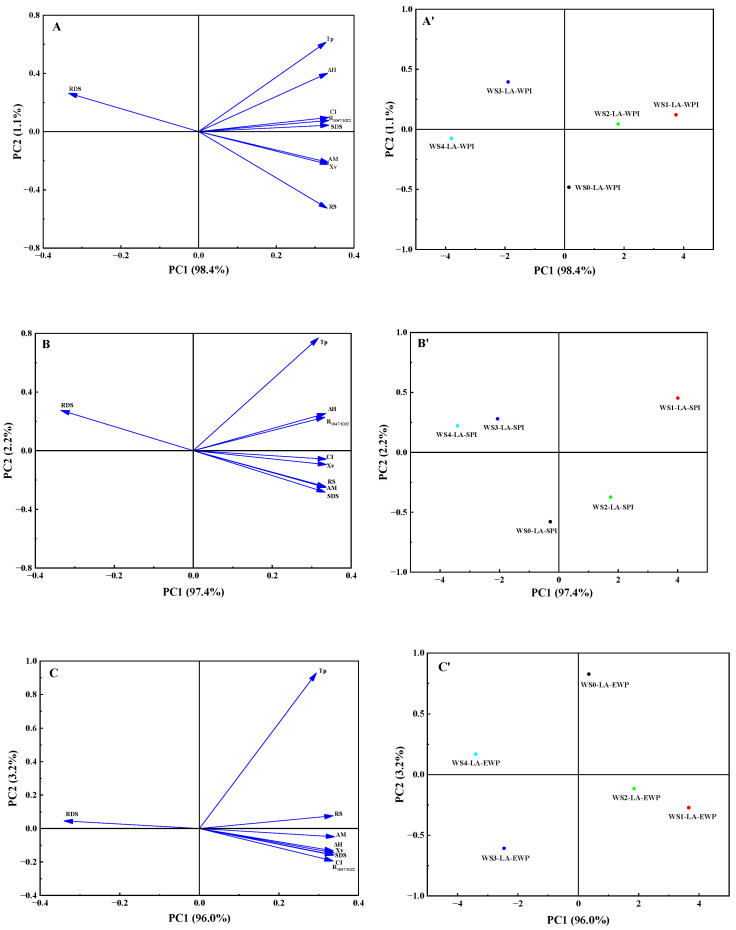
PCA of the structure and properties of different WS-LA-protein complexes: loading plot of WS-LA-WPI (**A**), WS-LA-SPI (**B**), and WS-LA-EWP (**C**); scoring plot of WS-LA-WPI (**A′**), WS-LA-SPI (**B′**), and WS-LA-EWP (**C′**).

PCA is a multivariate statistical analysis method that condenses the information of a group of interrelated indicators into a few principal components. It simplifies the dataset, reduces the number of variables, and helps researchers better understand the internal structure and law of the data [42]. As displayed in Figure 5, the loading plots and score plots of principal component analysis were used to analyze the multiple relations among the characteristic indexes of WS-LA-protein ternary complexes obtained by different ACP processing times. The cumulative contribution of PC1 and PC2 to the total variance was more than 99.0% of these three types of WS-LA-protein ternary complexes, indicating that PC1 and PC2 could reflect the principal information features of the obtained complexes comprehensively and effectively [20]. Loading plots are the reflection of the contribution of each variable (e.g., ΔH, CI, R_1047/1022_, amylose content) to the principal components. The direction of the arrow represents the direction of the effect on each component, and the length of the vector indicates the strength of the effect. Score plots provided the information on the differences between WS (0–4)-LA-protein ternary complexes. The distance between two variables reflects how similar they are in their properties. The closer the distance, the higher the similarity. For the loading plot of WS-LA-WPI complexes (Figure 5A), PC1 exhibited a positive correlation with T_p_, ΔH, R_1047/1022_, CI, Xv, amylose, SDS, and RS content and displayed a negative correlation with RDS content. PC2 exhibited a positive correlation with RDS content, T_p_, ΔH, R_1047/1022_, CI, and SDS content while presenting a negative correlation with Xv, amylose, and RS content. The above variables had little difference in their impact on PC1, while T_p_ and RS content have a significantly higher impact on PC2 than other variables. For the score plot of WS-LA-WPI complexes (Figure 5A’), these five samples were far apart, indicating that their structural and physicochemical properties were significantly different due to the different plasma treatment times of WS. WS0-LA-WPI was far away from the other four ternary complexes, indicating that ACP pretreatment could significantly affect the formation, structure, and properties of ternary complexes. For the WS-LA-SPI complexes, the loading plot (Figure 5B) was similar to that of WS-LA-WPI complexes; the only difference was that the CI and SDS content was negatively correlated to PC2. The score plot of WS-LA-SPI complexes (Figure 5B’) indicated that there were obvious distinctions in the structural and physicochemical properties among these WS (0–4)-LA-SPI complexes. In addition, WS1-LA-SPI was far away from another four ternary complexes, especially WS0-LA-SPI, meaning that ACP treatment of WS for 1 min could significantly affect the generation and physicochemical properties of WS-LA-SPI complexes. The distance between WS3-LA-SPI and WS4-LA-SPI was the closest, indicating that ACP processing on WS for 3 and 4 min had few effects on the structural properties of WS-LA-SPI complexes. For the WS-LA-EWP complexes, the loading plot (Figure 5C) explained that these variables had almost the same degree of influence on PC1, while T_p_ had a significantly higher degree of influence on PC2 than other variables. The score plot (Figure 5C’) showed that WS3-LA-EWP was farthest from WS0-LA-EWP, followed by WS1-LA-EWP, signifying that ACP treatment of WS for 1 and 3 min had the most obvious effect on physicochemical properties of WS-LA-EWP complexes. In general, ACP pretreatment of WS for different times could significantly impact the generation and structure of ternary complexes, regardless of the type of protein used in complexation.

## 4. Conclusions

In this study, the effect of different treatment times of ACP for WS on the formation, structure, and properties of ternary complexes among WS, LA, and different proteins (WPI, SPI, and EWP) was investigated. The experimental results indicated that ACP treatment on WS for 1 min might depolymerize amylopectin, release more linear chains, and increase the amylose content, which generated a larger number of ternary complexes, resulting in more ordered structure and lower digestibility. However, excessive ACP treatment (more than 1 min) could further depolymerize amylose chains into soluble oligosaccharides and decrease the amylose content, which caused a reduction in the number and order of the formed complexes and an increase in digestibility. Notably, the type of protein also affected the formation and properties of ternary complexes. For the WS treated by ACP for the same time, WPI formed a larger number, more ordered structure, and higher thermal stability of the ternary complex than SPI and EWP. This is because compared to SPI and EWP, WPI has a higher emulsifying property, leading to easier contact between starch and fatty acids and stronger hydrogen bonds between starch and protein. In summary, ACP pretreatment can regulate and control the formation and structure of ternary complexes, which provides a theoretical basis for expanding the application of complexes, such as special medical food and low-GI food.

## Figures and Tables

**Table 1:** Amylose content of ACP-pretreated WS.

Samples	AM (%)
WS0	28.71 ± 0.22 ^c^
WS1	30.02 ± 0.19 ^a^
WS2	29.29 ± 0.08 ^b^
WS3	27.71 ± 0.10 ^d^
WS4	27.23 ± 0.21 ^e^

Values are means ± SD. Values with the different letters in the same column are significantly different (*p* < 0.05).

**Table 2:** The CI, crystalline structure, short-range ordered structure, and thermal properties of different WS-LA-protein complexes.

Samples	CI (%)	Xv (%)	R_1047/1022_	T_o_ (°C)	T_p_ (°C)	T_c_ (°C)	ΔH (J/g)
WS0-LA-WPI	68.33 ± 0.11 ^b^	10.18 ± 0.07 ^c^	0.798 ± 0.001 ^c^	91.26 ± 0.10 ^a^	99.58 ± 0.15 ^a^	106.67 ± 0.08 ^ab^	3.57 ± 0.11 ^c^
WS1-LA-WPI	69.21 ± 0.19 ^a^	10.56 ± 0.06 ^a^	0.811 ± 0.002 ^a^	91.49 ± 0.09 ^a^	99.82 ± 0.11 ^a^	107.02 ± 0.14 ^a^	4.26 ± 0.19 ^a^
WS2-LA-WPI	68.62 ± 0.16 ^b^	10.39 ± 0.07 ^b^	0.804 ± 0.001 ^b^	91.60 ± 0.20 ^a^	99.74 ± 0.06 ^a^	106.75 ± 0.16 ^ab^	3.93 ± 0.13 ^b^
WS3-LA-WPI	67.91 ± 0.10 ^c^	9.87 ± 0.04 ^d^	0.792 ± 0.002 ^d^	91.19 ± 0.19 ^a^	99.56 ± 0.21 ^a^	106.45 ± 0.11 ^b^	3.42 ± 0.08 ^cd^
WS4-LA-WPI	67.34 ± 0.12 ^d^	9.72 ± 0.06 ^e^	0.787 ± 0.003 ^e^	91.01 ± 0.18 ^a^	99.38 ± 0.06 ^a^	106.37 ± 0.10 ^b^	3.14 ± 0.14 ^d^
WS0-LA-SPI	65.94 ± 0.14 ^c^	9.93 ± 0.05 ^c^	0.784 ± 0.002 ^b^	92.53 ± 0.29 ^b^	98.6 ± 0.24 ^a^	106.72 ± 0.15 ^a^	3.36 ± 0.18 ^ab^
WS1-LA-SPI	67.41 ± 0.19 ^a^	10.13 ± 0.06 ^a^	0.792 ± 0.001 ^a^	92.98 ± 0.21 ^ab^	99.07 ± 0.21 ^a^	106.82 ± 0.14 ^a^	3.73 ± 0.23 ^a^
WS2-LA-SPI	66.73 ± 0.23 ^b^	10.02 ± 0.07 ^b^	0.786 ± 0.002 ^b^	93.20 ± 0.09 ^a^	98.80 ± 0.13 ^a^	106.71 ± 0.25 ^a^	3.53 ± 0.15 ^ab^
WS3-LA-SPI	65.36 ± 0.16 ^d^	9.82 ± 0.04 ^d^	0.781 ± 0.003 ^c^	92.51 ± 0.24 ^b^	98.64 ± 0.14 ^a^	106.65 ± 0.11 ^a^	3.28 ± 0.13 ^b^
WS4-LA-SPI	64.78 ± 0.22 ^e^	9.76 ± 0.07 ^e^	0.779 ± 0.002 ^d^	92.41 ± 0.19 ^b^	98.53 ± 0.16 ^a^	106.56 ± 0.08 ^a^	3.19 ± 0.05 ^b^
WS0-LA-EWP	61.31 ± 0.10 ^c^	9.14 ± 0.04 ^b^	0.757 ± 0.001 ^c^	90.23 ± 0.11 ^a^	97.78 ± 0.13 ^a^	105.57 ± 0.22 ^a^	2.69 ± 0.15 ^bc^
WS1-LA-EWP	62.81 ± 0.23 ^a^	9.43 ± 0.07 ^a^	0.770 ± 0.002 ^a^	90.29 ± 0.16 ^a^	97.77 ± 0.12 ^a^	105.65 ± 0.09 ^a^	3.13 ± 0.14 ^a^
WS2-LA-EWP	62.19 ± 0.19 ^b^	9.32 ± 0.05 ^ab^	0.762 ± 0.001 ^b^	90.61 ± 0.30 ^a^	97.69 ± 0.19 ^a^	105.77 ± 0.08 ^a^	2.95 ± 0.13 ^ab^
WS3-LA-EWP	60.38 ± 0.15 ^d^	8.97 ± 0.04 ^c^	0.751 ± 0.002 ^d^	90.32 ± 0.16 ^a^	97.25 ± 0.13 ^b^	105.45 ± 0.11 ^a^	2.43 ± 0.09 ^cd^
WS4-LA-EWP	59.51 ± 0.17 ^e^	8.86 ± 0.07 ^d^	0.748 ± 0.001 ^e^	90.16 ± 0.13 ^a^	97.37 ± 0.21 ^ab^	105.36 ± 0.21 ^a^	2.29 ± 0.12 ^d^

Values are means ± SD. Values with the different letters for the same protein complexes in the same column are significantly different (*p* < 0.05).

## Data Availability

The data presented in this study are not publicly available at this time but can be obtained from the first author upon a reasonable request.
